# Experimental Study of Hot-Sphere Anemometer Response in Stratospheric Environment

**DOI:** 10.3390/s24206674

**Published:** 2024-10-17

**Authors:** Xiyuan Li, Xiaoning Yang, Xiaobin Shen, Guiping Lin, Dongxing Tao, Jing Wang

**Affiliations:** 1 Beijing Institute of Spacecraft Environment Engineering, Beijing 100094, China; lxy_422@msn.com (X.L.); yxn_xxlw@163.com (X.Y.); 2 School of Aeronautic Science and Engineering, Beihang University, Beijing 100191, China; shenxiaobin@buaa.edu.cn (X.S.); gplin@buaa.edu.cn (G.L.); 3 Hangzhou International Innovation Institute, Beihang University, Hangzhou 311115, China; 4 China Aerospace Science and Technology Corporation, Beijing 100048, China; wangjing19800510@buaa.edu.cn

**Keywords:** stratosphere, wind speed measurement, experiment, thermal anemometer

## Abstract

Accurate wind speed measurement in low-pressure conditions is crucial for the thermal performance validation and attitude control of stratospheric aircraft. As air density decreases, traditional wind speed measurement systems based on principles such as dynamic pressure, heat transfer, ultrasound, and particle velocimetry face significant challenges when applied in low-pressure environments, often failing to achieve the required measurement accuracy. This paper presents the development of a wind speed simulation system based on a rotation method designed to operate in low-pressure conditions, utilizing a space environment simulation chamber in conjunction with a high-precision turntable. The system was employed to conduct response tests on a constant heat flow thermal sphere anemometer within a stratospheric pressure range of 1 kPa to 30 kPa. The experimental results revealed that at extremely low Reynolds numbers, the probe signal exhibited increasing nonlinearity, significantly affecting the response curve at pressures below 15 kPa. While the sensitivity of the hot-sphere probe remained relatively stable at wind speeds above 5 m/s, it decreased nonlinearly as the pressure dropped when wind speeds fell below 5 m/s. Furthermore, this paper analyzes the impact of various interpolation methods on wind speed conversion errors, providing valuable data to support the future development and validation of stratospheric aircraft.

## 1. Introduction

Stratospheric low-speed aircraft, such as stratospheric airships and high-altitude, long-endurance, unmanned aerial vehicles (UAVs), possess significant strategic value in areas such as environmental monitoring, Earth observation, emergency communications, and infrared early warning. These aircraft typically operate at altitudes between 10 km and 30 km [1,2]. Recent advancements in foundational sciences and engineering technologies, including in lightweight and high-strength materials, efficient solar cells, high-energy-density batteries, and high-efficiency propulsion systems, have enabled the development of various types of stratospheric aircraft in numerous countries and regions [3].

The stratosphere is characterized by low temperatures and low pressure. For instance, at an altitude of 10 km, the temperature can drop to −50 °C, with a pressure of approximately 26 kPa, while, at 30 km, the temperature is around −46 °C, and the pressure falls to as low as 1 kPa. The low temperatures pose the risk of component failure for stratospheric aircraft when they are not operational [4], and the low pressure significantly reduces convective heat transfer, potentially leading to component failure due to inadequate heat dissipation during high-power operation [5,6]. Therefore, precise thermal design and validation are critical for stratospheric aircraft [7,8]. However, constructing relevant validation environments depends on the accurate simulation and measurement of wind speed in low-pressure conditions. Moreover, accurate wind speed data are crucial for the attitude control of stratospheric airships [9,10]. Consequently, accurate wind speed measurement in low-pressure conditions is a key challenge in the development and validation of stratospheric aircraft.

The extremely low pressure in the stratosphere leads to significant changes in air density, thermal conductivity, and other parameters, making it difficult for commonly used anemometers to accurately measure low wind speeds in the stratospheric environment. For dynamic pressure-based anemometers, such as Pitot tubes and five-hole probes, the reduction in air density leads to a substantial decrease in dynamic pressure. At an altitude of 30 km, the air density is only about 1% of its value at sea level, and the dynamic pressure generated by a wind speed of 5 m/s is only 0.2 Pa, requiring highly sensitive micro-manometers for measurement. This complexity results in a bulky system, making it challenging to implement for multi-point wind speed measurements in ground tests [11,12]. The cup anemometer features advantages such as a simple structure, excellent linearity, and ease of calibration. Recent research indicates that after optimization, the operational ceiling of the cup anemometer can reach altitudes of up to 25 km. Current primary issues include slow response time and increased axial friction [13]. Although ultrasound-based wind speed measurement is less affected by pressure changes, the sound transmission capability in a 1 kPa environment is less than 1% of its capability at standard atmospheric pressure, making it difficult to distinguish ultrasound signals from noise [14]. Additionally, the large size of these devices renders them unsuitable for multi-point wind speed monitoring in experiments [15]. Laser Doppler anemometry (LDA) and particle image velocimetry (PIV) systems currently face challenges in miniaturization. Although these systems can measure tracer particle velocities with a resolution on the order of 0.01 m/s, reduced particle tracking accuracy under low pressure leads to a speed discrepancy of over 5% between the fluid and tracer particles in regions with significant flow velocity variations [16,17], making it difficult to accurately measure complex flow fields under 1 kPa conditions [18]. Thermal anemometers, including hot-wire, hot-film, and hot sphere, are extensively used in experimental setups such as wind tunnels. Hot-wire and hot-film probes with micrometer-scale characteristic lengths, driven by constant temperature anemometer (CTA) systems, can measure wind speeds across a broad range, from low velocities to supersonic speeds. These probes are also capable of capturing high-frequency velocity fluctuations in small-scale regions of the flow field, making them essential for measuring turbulence parameters in fluid dynamics. Additionally, due to their simple design and high reliability, custom-built thermal anemometers are widely applied in specialized, low-pressure environments, such as for wind speed measurements on Mars [19,20]. However, the primary challenge lies in the nonlinear decrease in sensor output signal caused by the reduced heat transfer capability of air in low-pressure environments [21,22], as well as measurement errors due to resistance changes induced by low temperatures [23]. While these devices can achieve excellent accuracy and response times after calibration, precise knowledge of environmental conditions is required for calibration [24,25].

In summary, currently available wind speed measurement techniques are difficult to apply directly under the extremely low pressure of the stratosphere, particularly for real-time, high-precision, multi-point measurements of complex wind speeds in experimental settings. This research, based on the principles of thermal anemometry and using a typical hot-sphere anemometer as the object of study, conducted tests on the sensor’s output response under different pressures by simulating the relative motion between the hot-sphere probe and the flow field using a space environment simulator and a high-precision turntable. The feasibility of stratospheric wind speed measurement based on hot-sphere anemometers is analyzed, providing support for the future development, validation, and testing of stratospheric aircraft.

## 2. Experimental System

### 2.1. Thermal Anemometer

The thermal anemometry method measures airflow speed by assessing the relationship between convective heat transfer intensity and flow speed. Thermal anemometers can be classified into hot-wire, hot-film, and hot-sphere types based on the probe design. From a measurement principle perspective, thermal anemometry can be divided into constant temperature and constant heat flow methods. In the former, the probe is maintained at a constant temperature, and airflow speed is determined by measuring the power or voltage applied. In contrast, the latter method involves applying a constant heat flow to the probe and measuring the probe’s temperature to characterize airflow speed [26,27].

In order to achieve a higher output signal for the anemometer, the sensor should be operated in a continuous flow regime with a high convective heat transfer coefficient. According to the definition of the Knudsen number (Kn) [28,29]:

(1) $$Kn=\frac{\lambda }{L}$$

where L is the characteristic length of the probe (m) and λ is the mean free path of gas molecules (m), which can be expressed as follows:

(2) $$\lambda =\frac{1}{\sqrt{2}\pi d^{2}n_{0}}=\frac{RT}{\sqrt{2}\pi d^{2}N_{A}P}$$

**Figure 1:**
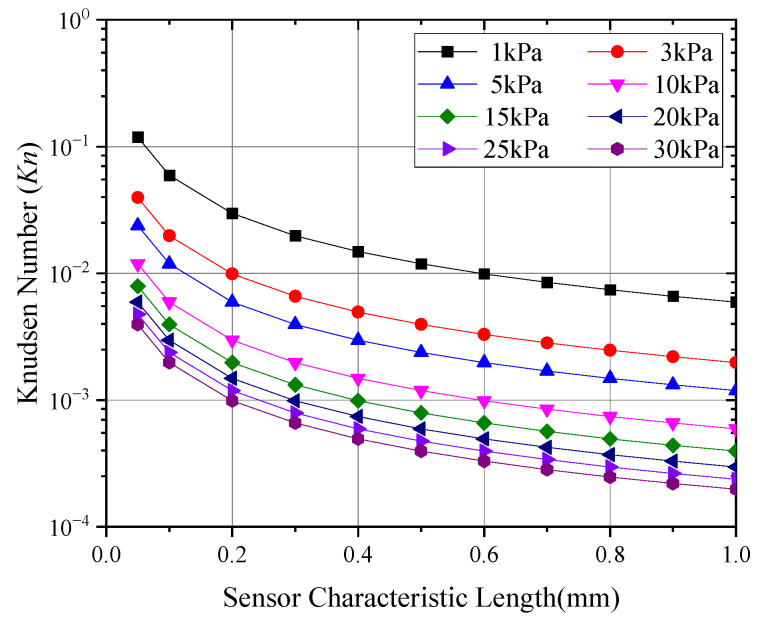
Knudsen number of wind speed probes under low pressure.

In this equation, d represents the molecular kinetic diameter (m) and $n_{0}$ is the number density of molecules per unit volume. The value of $n_{0}$ can be calculated based on the ambient temperature T(K), pressure P(Pa), gas constant R(8.3145 J/mol·K), and Avogadro’s number $N_{A}$($6.0221\times 10^{23}\;{mol}^{-1}$). The molecular kinetic diameter of air is approximately 0.364 nm. Figure 1 shows the Knudsen number of probes with different characteristic lengths in a low-pressure environment.

**Figure 2:**
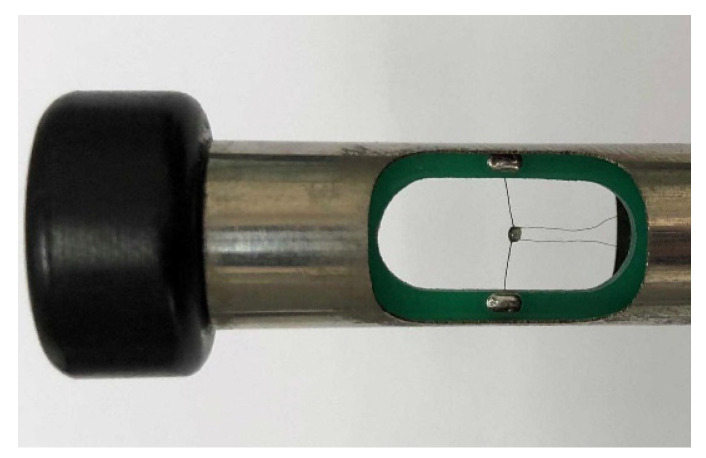
Hot-sphere anemometer.

**Figure 3:**
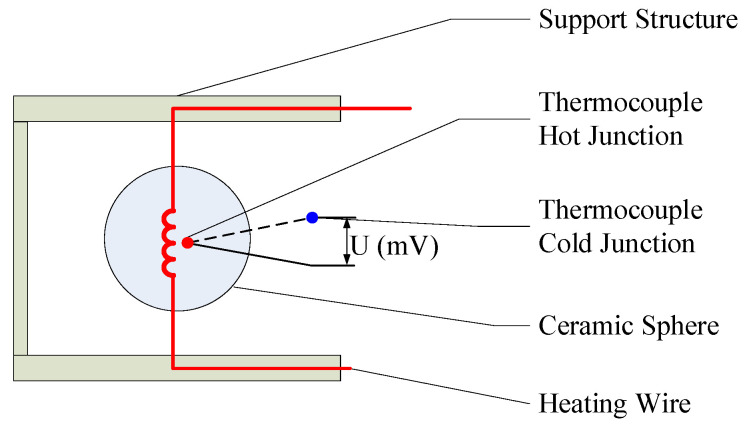
Schematic diagram of hot-sphere anemometer.

As observed in Figure 1, to ensure that a wind speed probe operates in the continuous flow regime, the characteristic length of the probe should be no less than 0.6 mm when measuring wind speeds in environments with pressures as low as 1 kPa. Given considerations such as heat transfer and long-distance signal transmission, this study focuses on the relatively simple constant-heat-flow hot-sphere anemometer as a typical research subject. The core of this device is a small, ceramic-encased sphere with a diameter of approximately 0.8 mm. The internal temperature-sensing element is the hot junction of a Type E thermocouple, while the heating element is a stable resistance constantan wire, with a total resistance of approximately 50 Ω, wherein the internal and external components of the hot sphere account for roughly 50% each. The hot sphere also includes the cold junction of the thermocouple externally [30,31]. During operation, a near-constant heat flow is applied to the thermocouple wire via a constant current or constant voltage power supply. As the airflow speed increases, the temperature of the hot sphere gradually approaches that of the incoming air. The electromotive force between the hot and cold junctions of the thermocouple reflects the temperature difference between the hot sphere and the fluid. Through calibration, a conversion relationship between the probe’s electromotive force and the airflow speed can be established. Figure 2 and Figure 3 illustrate a hot-sphere probe and its schematic diagram, respectively.

In the experiments, a constant current applied to the heating wire generated a steady power output. Under these conditions, the cold junction temperature of the thermocouple could be regarded as the fluid temperature, while the hot junction temperature corresponded to that of the hot sphere. Consequently, the heat flow on the hot sphere could be expressed as

(3) $${{Q\_S}_{sphere}=Q\_Conv}_{sphere}+{Q\_Rad}_{sphere}+{Q\_Cond}_{Tc1}+{Q\_Cond}_{Tc2}+{Q\_Cond}_{HW}$$

In this equation, ${Q\_S}_{sphere}$ represents the heat generated within the ceramic sphere (W); ${Q\_Conv}_{sphere}$ denotes the heat transferred from the ceramic sphere to the environment via convection (W); ${Q\_Rad}_{sphere}$ signifies the heat radiated from the ceramic sphere to the environment (W); ${Q\_Cond}_{Tc1}$ and ${Q\_Cond}_{Tc2}$ correspond to the heat conducted from the ceramic sphere to thermocouple wires 1 and 2, respectively (W); and ${Q\_Cond}_{HW}$ indicates the heat conducted from the ceramic sphere to the external heating wire (W). The conductive terms ${Q\_Cond}_{Tc1}$, ${Q\_Cond}_{Tc2}$, and ${Q\_Cond}_{HW}$ are related to the operating temperatures of the thermocouple wires and the heating wire. Assuming that their operating temperatures vary in the same manner as that of the ceramic sphere and that the proportion of heat conduction remains approximately constant, Equation (3) can be simplified as follows:

(4) $$a{{\times Q\_S}_{sphere}=Q\_Conv}_{sphere}+{Q\_Rad}_{sphere}$$

In this equation, a represents the percentage of heat generated within the ceramic sphere that is transferred to the environment via convection and thermal radiation (%).

Expanding the terms for the heat transferred from the ceramic sphere to the environment by convection ${Q\_Conv}_{sphere}$ and thermal radiation ${Q\_Rad}_{sphere}$ leads to Equation (5):

(5) $${a\times Q\_S}_{sphere}=s_{sphere}h\left(T_{sphere}-T_{e}\right)+s_{sphere}\varepsilon_{sphere}\sigma \left({T_{sphere}}^{4}-{T_{e}}^{4}\right)$$

In this equation, $s_{sphere}$ represents the surface area of the ceramic sphere ($m^{2}$), $T_{sphere}$ is the average temperature of the ceramic sphere (K), and $T_{e}$ is both the ambient temperature and the radiation background temperature (K). The term $\varepsilon_{sphere}$ denotes the surface emissivity of the ceramic sphere, while σ is the Stefan–Boltzmann constant ($5.67\times 10^{-8}\;W/(m^{2}\cdot K^{4}$)). The coefficient h represents the convective heat transfer coefficient at the surface of the ceramic sphere ($W/(m^{2}\cdot K)$), which can be expressed as a monotonically increasing function of airflow speed, $f_{h}(V)$. Therefore, when the ambient temperature and pressure are stable, a specific airflow speed V corresponds to a particular hot sphere temperature $T_{sphere}$. By measuring the thermoelectric potential between the cold and hot junctions of the thermocouple, the current airflow speed can be characterized.

### 2.2. Experimental System Based on Rotation Method

To test the response of the hot-sphere anemometer under low-pressure conditions, it was essential to generate a uniform, stable, and well-characterized airflow field within a low-pressure environment. However, current wind tunnel facilities face challenges in creating continuous airflow at extremely low pressures, making it necessary to rely on space environment simulation chambers to establish a stable, low-pressure environment. Feasible technical approaches include the rotational method, the closed-loop wind tunnel method, and the open-circuit wind tunnel method.

The closed-loop wind tunnel method uses a fan to generate stable airflow within the space environment simulation chamber; however, it lacks a standardized wind speed source, necessitating calibration with higher-precision anemometers [32]. The open-circuit wind tunnel method drives airflow by utilizing the pressure differential between the inside and outside of the simulation chamber, with vacuum pump control to maintain stable pressure. The main drawbacks of this method include poor uniformity in simulating low-speed airflow and the complexity of control [33]. The rotation method simulates relative motion by using a turntable to create airflow near the probe. However, higher rotational speeds may induce resonance of the rotating arm, thereby affecting the operation of the experimental system [34].

**Figure 4:**
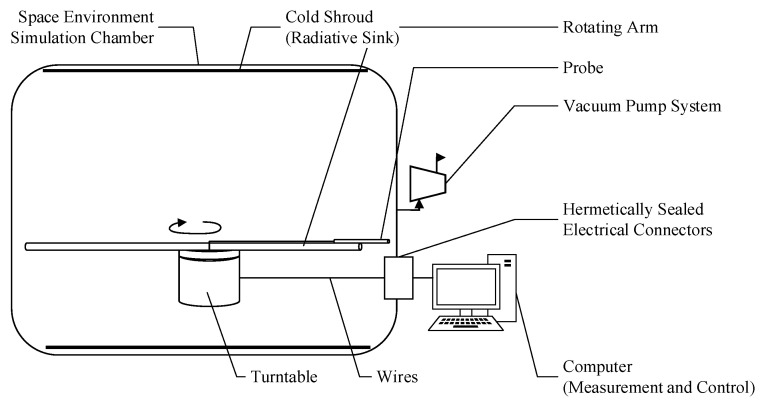
Simulation schematic diagram.

**Figure 5:**
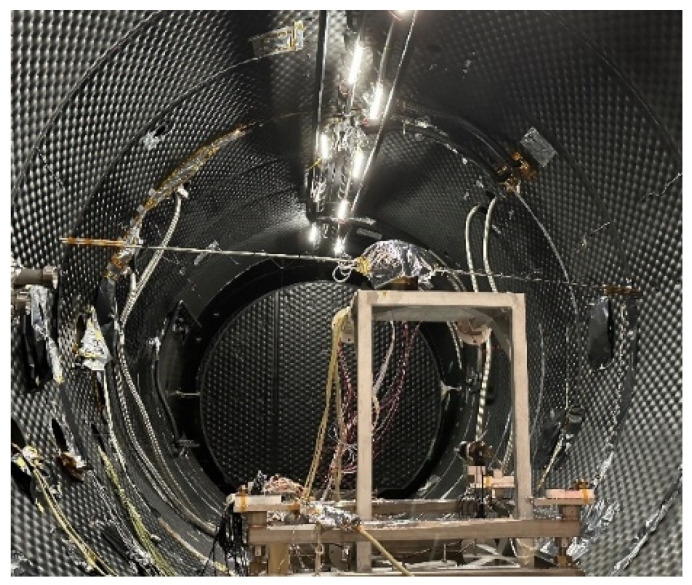
Simulation system.

In this study, we developed a testing apparatus for the rotational wind speed simulation method using a space environment simulation chamber. The apparatus involved rotating the anemometer probe via a turntable and rotating arm within the chamber, thereby simulating airflow at low pressure through the relative motion between the probe and the gas. The schematic diagram and experimental setup are shown in Figure 4 and Figure 5, respectively.

As shown in Figure 5, the KM3E space environment simulation chamber had an available diameter of 3.6 m. The internal pressure of the chamber was precisely controlled to an accuracy of ±50 Pa through the use of a vacuum pump system and an aperture control valve for evacuation and refilling. Temperature control was achieved by multi-channel electric heaters that precisely regulated the temperature of cold nitrogen gas, enabling accurate control of both the chamber’s radiative heat exchanger (cold sink) and the gas temperature, with a control accuracy of ±3 °C.

A high-precision turntable was installed at the center of the space environment simulation chamber, with a rotational arm length of approximately 2.5 m. The turntable was driven by a controller and computer system located outside the chamber, providing a rotational speed accuracy of ±1 RPM. The hot-sphere probe was mounted at the end of the rotational arm, positioned about 1.28 m from the center of rotation. During testing, the probe was rotated by the arm to simulate the airflow speed being measured by the probe. The data acquisition device for the hot-sphere anemometer (mV signal acquisition device) was also mounted on the high-precision turntable, allowing for in situ collection of the millivolt-level weak signals.

Additionally, the space environment simulation chamber was equipped with external hardware, including a computer and programmable power supply. Through hermetically sealed electrical connectors, the hot-sphere probe and data acquisition equipment were powered, and digital signals from the acquisition device were decoded, displayed, and stored.

### 2.3. Measurement and Control System

**Figure 6:**
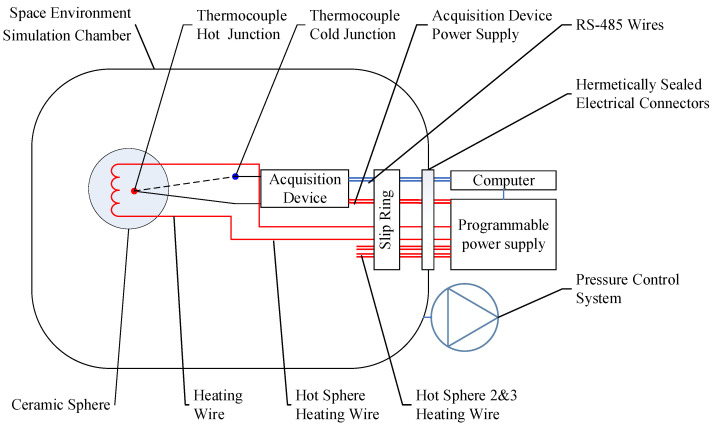
Measurement and control system.

To drive the probes mounted on the rotating arm, a constant current needed to be applied to the probes and the millivolt-level electromotive force of the thermocouples on the probes needed to be measured. Due to the limitations of the thermocouple measurement principle, differences in temperature between various metals used in the measurement chain, such as slip rings and electrical connectors, can introduce additional errors. Additionally, long-distance signal transmission can lead to signal interference and loss. Therefore, it was essential to collect and transmit the millivolt signals at the slip ring, converting them into digital signals for transmission to an external space environment simulation container, where they could be read and stored by an external computer. Figure 6 illustrates the connection of the measurement system.

The millivolt measurement device on the turntable was a custom KBM-309G millivolt signal acquisition device, with a measurement range of 0 to 75 mV and a resolution of 12 bits, resulting in a minimum resolution of 18 μV. Its basic parameters are detailed in Table 1.

## 3. Experimental Results and Analysis

### 3.1. Test Conditions

**Figure 7:**
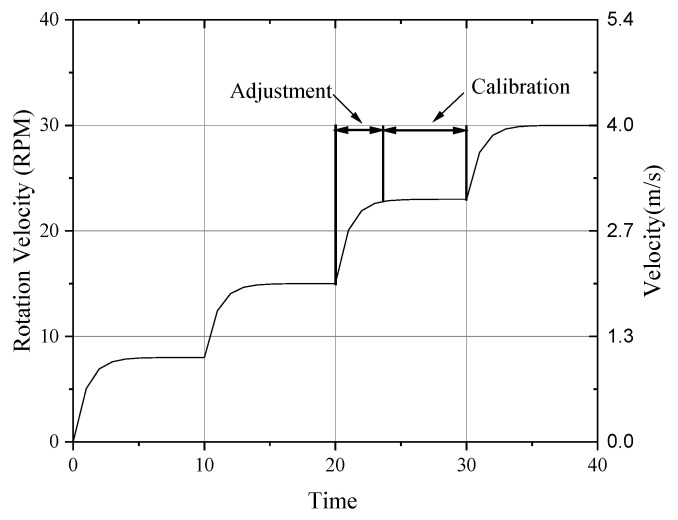
Illustration of wind speed application.

The thermal anemometer probe measured the temperature difference between the hot and cold junctions of its thermocouple to determine the temperature difference between the thermal sphere and the ambient environment. Due to the utilization of temperature difference measurement methods, the signal from the hot-sphere probe was less affected by environmental temperature and, relatively, more influenced by environmental pressure [35]. Consequently, this study selected a constant temperature of −20 °C for testing, with experimental pressures set at 1 kPa, 3 kPa, 5 kPa, 10 kPa, 15 kPa, 20 kPa, 25 kPa, and 30 kPa, corresponding to altitudes ranging from approximately 9 km to 31 km. During the experiments, a constant current of 45 mA was applied to the thermal sphere. For each experimental pressure, wind speeds ranging from 0 to 25 m/s were incrementally applied at rotational speeds of 0 to 188 RPM, as illustrated in Figure 7. After stabilizing the rotational speed (within ±0.5 RPM of the target), the probe was calibrated.

**Figure 8:**
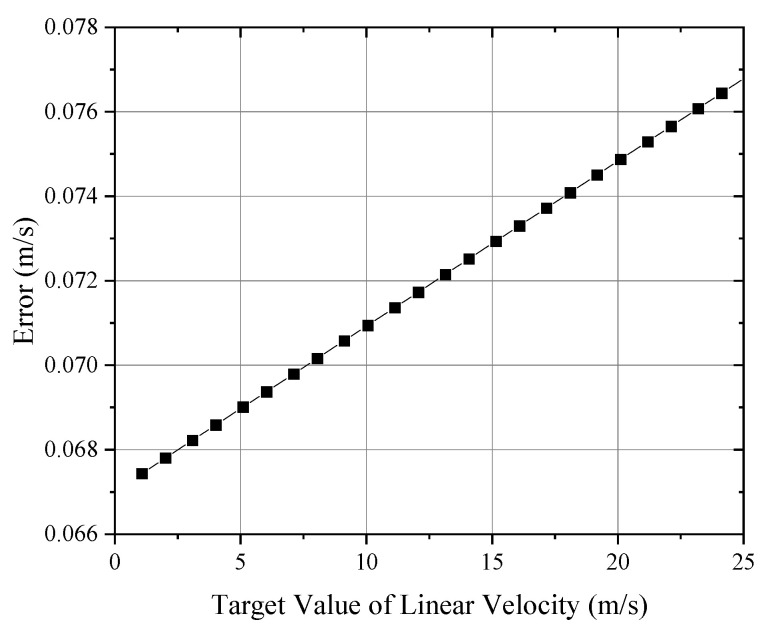
Simulated velocity error.

According to the principle of the rotational calibration method, the linear velocity V can be expressed as the product of the angular velocity ω and the rotational radius R. Thus, the linear speed error of the turntable was composed of the measurement error of the arm diameter and the error in the rotational speed:

(6) dV=ωdR+Rdω

where dV is the simulated relative speed error (m/s); ω is the current angular velocity of the turntable (rad/s); R is the rotational radius (1.28 m); dR is the measurement error of the radius (m), with an uncertainty of 0.5 mm, as measured by a ruler; and dω is the angular velocity measurement error (rad/s), with a device-measured error of 0.5 RPM. Substituting these values allowed for the calculation of the system’s simulated linear velocity error. As shown in Figure 8, for linear velocities up to 25 m/s, the simulation error remained below 0.08 m/s.

### 3.2. Natural Convection Analysis

To assess the impact of natural convection on the surface of the thermal sphere, the Richardson number (Ri) analysis method was introduced. When Ri is below 0.01, forced convection dominates; when it exceeds 10, natural convection is dominant; and in the intermediate range, mixed convection must be considered [36]:

(7) $$Ri=\frac{Gr}{{Re}^{2}}=\frac{\frac{ga_{v}∆Tl^{3}}{\upsilon^{2}}}{{\left(\frac{Vl}{\upsilon }\right)}^{2}}=\frac{ga_{v}∆Tl}{V^{2}}$$

where Gr is the Grashof number, Re is the Reynolds number, g is the gravitational acceleration (${m/s}^{2}$), υ is the kinematic viscosity of the gas ($m^{2}/s$), l is the characteristic length (m), ∆T is the temperature difference between the surface and the environment (K), V is the freestream speed (m/s), and $a_{v}$ is the volumetric thermal expansion coefficient of the gas ($K^{-1}$), which can be expressed as

(8) $$a_{v}=-\frac{1}{\rho }{\left(\frac{\partial \rho }{\partial T}\right)}_{P}=-\frac{1}{\rho }{\left(\frac{\partial \left(\frac{P}{RT}\right)}{\partial T}\right)}_{P}=-\frac{1}{\rho }\times \frac{P}{R}\times (-\frac{1}{T^{2}})=\frac{1}{T}$$

where ρ is the density ($kg/m^{3}$), P is the pressure (Pa), R is the specific gas constant, and T is the absolute temperature (K). Therefore, in Equations (7) and (8), the gravitational acceleration g is constant, the characteristic length l is calculated based on the thermal sphere diameter of 0.8 mm, and the temperature difference ∆T and absolute temperature T are calculated from the thermocouple output voltage U, the thermoelectric coefficient a (Seebeck coefficient), and the ambient temperature $T_{e}$, as shown in Equation (9):

(9) $$∆T=U/a\;T=T_{e}+∆T/2$$

**Figure 9:**
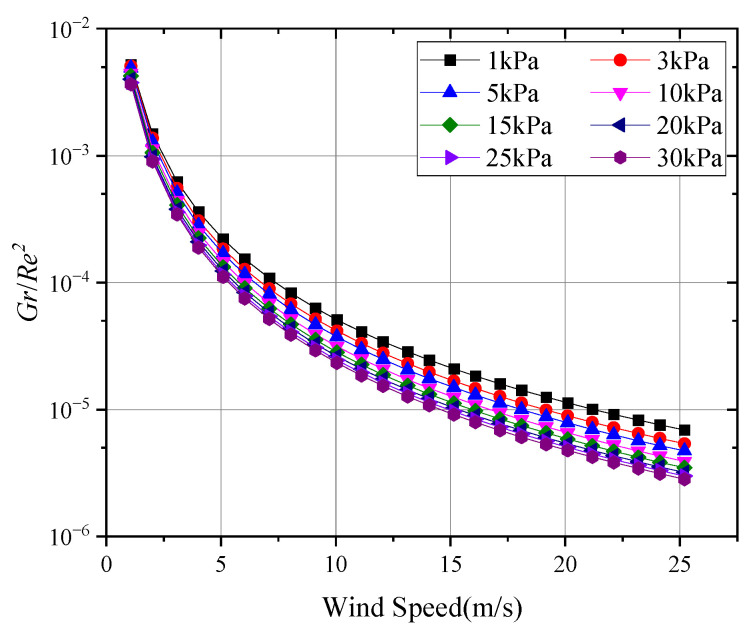
Richardson numbers Ri for thermal probes under different conditions.

Thus, when the gravitational acceleration and geometric characteristics are known, the Richardson number Ri can be determined from the absolute temperature T, the temperature difference ∆T, and the freestream speed V. The Richardson numbers Ri for the thermal anemometer probe under different pressures and wind speeds are shown in Figure 9.

As depicted in the figure, within the pressure range of 1 kPa to 30 kPa tested in this study, when the wind speed exceeded 1 m/s, the convective heat transfer on the surface of the thermal anemometer probe could still be regarded as forced convection.

### 3.3. Response of Hot-Sphere Probe

**Figure 10:**
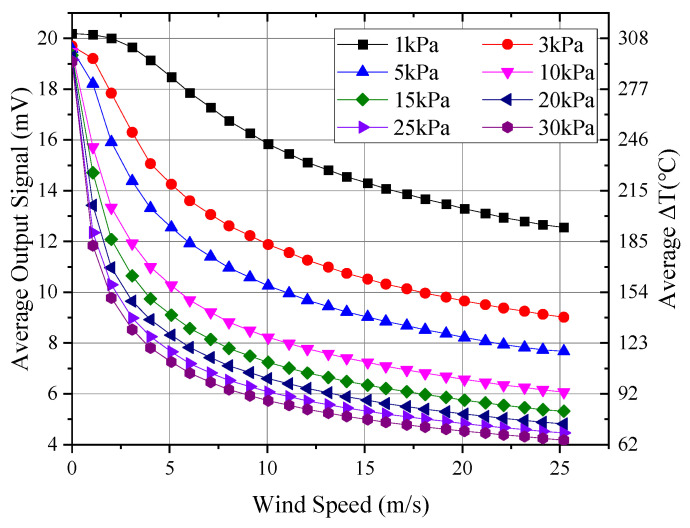
Average response at different pressures.

**Figure 11:**
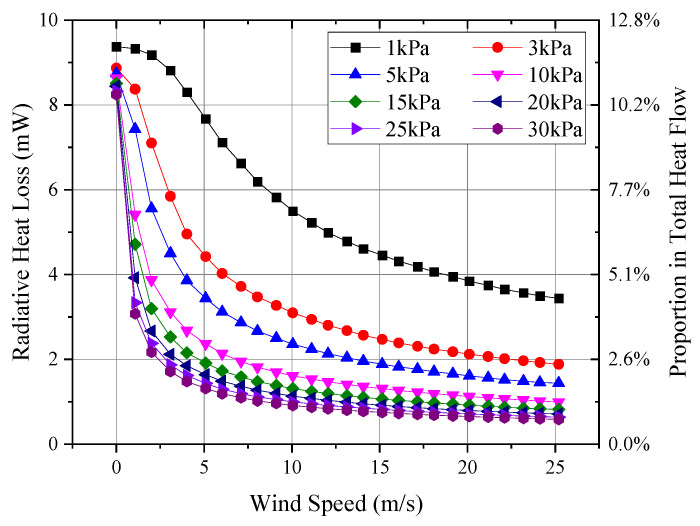
Radiative heat loss.

Through a series of low-pressure tests involving three thermal anemometer probes, Figure 10 shows the curves of probe output variation with wind speed under different pressures. The output signal shown represents the average of the three probes. The temperature difference ∆T between the thermal sphere and the environment was calculated using the probe’s output signal and the Seebeck coefficient of the internal thermocouple, which was approximately 0.065 mV/°C. Assuming an infrared emissivity of 0.85 for the surface of the hot sphere, the radiative heat loss could be calculated based on the temperature of the hot sphere, along with its proportion of the total heat flow. The total heat flow was determined from the hot sphere’s current of 0.056 mA and a sphere resistance of 25 Ω. Figure 11 presents the calculated radiative heat loss and its proportion relative to the total sphere heat flow.

As illustrated in Figure 10, the output curves of the probes exhibited notable similarity within the pressure range of 15 to 30 kPa, with the curves approximately shifting horizontally at wind speeds exceeding 5 m/s. However, at pressures below 15 kPa, the differences between the curves became progressively more pronounced. Specifically, the divergence between the 1 kPa and 3 kPa curves was comparable to that observed between the 3 kPa and 10 kPa curves. Additionally, as the pressure decreased, the shape of the curves also changed. Between 15 kPa and 30 kPa, the wind speed–signal curve was a purely concave function, with the second derivative of the curve being consistently greater than zero. As the pressure decreased to around 5 kPa, the wind speed curve began to transition to a function that was initially convex and then concave, exhibiting an inflection point where the second derivative equaled zero. The position of this inflection point shifted towards higher wind speeds as the pressure decreased, occurring at approximately 2 m/s, 3 m/s, and 5 m/s for 5 kPa, 3 kPa, and 1 kPa, respectively.

As shown in Figure 11, the constant-heat-flow hot-sphere probe experienced a temperature difference of approximately 200 °C under low and high wind speeds, resulting in significant variation in radiative heat loss. At low wind speeds, the radiative heat loss can exceed four times that at high wind speeds. However, the maximum radiative heat loss was approximately 10 mW, accounting for about 12% of the total heat flow of the hot sphere. Therefore, it did not significantly impact the signal characteristics of the probe.

**Figure 12:**
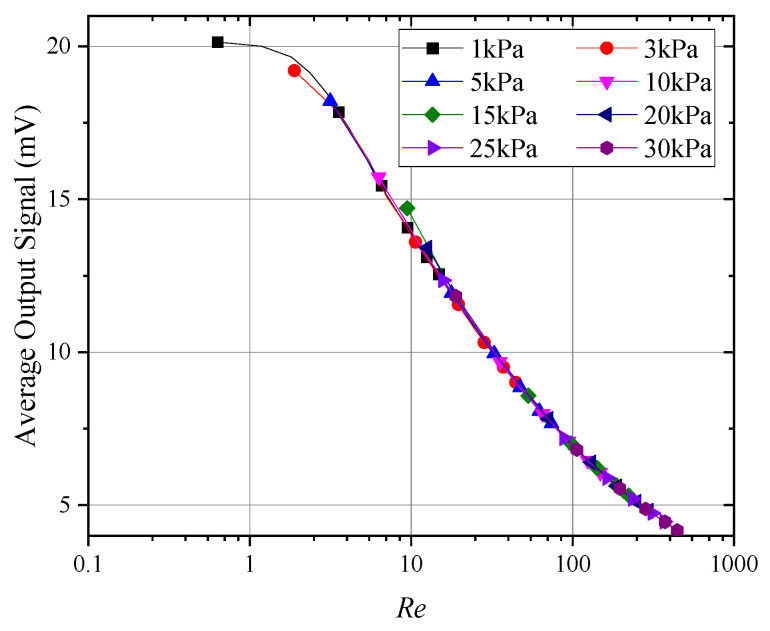
Average response at different Reynolds numbers.

Figure 12 presents the curves of the average output signal from hot-sphere probes with Reynolds numbers under different pressure conditions. As illustrated, the curves exhibit strong consistency across all segments, confirming the validity of the experimental methods and data processing employed in this study. Furthermore, when the Reynolds number dropped below 10, the signal–Reynolds number curve gradually became nonlinear. This behavior explains the nonlinear variations in the probe signal under low-pressure conditions, as shown in Figure 10. Therefore, for thermal anemometers operating in environments below 5 kPa, the calibration range must at least cover the full range of expected operating conditions. Extrapolation methods are unlikely to accurately simulate changes in the probe output curve, potentially introducing significant errors.

Based on Figure 12, the Nusselt number on the hot sphere could be estimated. Using Equation (5), the convective heat transfer coefficient h on the hot sphere could be calculated. The proportionality constant a, which depended on the specific design of the thermal sphere, heating wire, and thermocouple, was determined through data fitting to be approximately 0.7. This implied that 70% of the heat generated by the internal heating wire was dissipated via convection and radiation from the thermal sphere, while the remaining 30% was transferred by conduction to the connected thermocouple and heating wire, where it was further dissipated through convection and radiation. The Nusselt number could then be calculated from the convective heat transfer coefficient h:

(10) Nu=hl/λ

In this equation, l represents the characteristic length of the heated sphere (0.0008 m) and λ is the thermal conductivity of air (W/(m∙K)), which can be calculated based on temperature. Additionally, for low Reynolds number flow over the surface of a sphere, the Nusselt number can be calculated using the empirical correlation proposed by Finlayson [37]:

(11) $$\frac{1}{Nu-2}=\frac{1}{Pe/2}+\frac{1}{\left(0.9{Pe}^{1/3}{Re}^{0.11}\right)}$$

**Figure 13:**
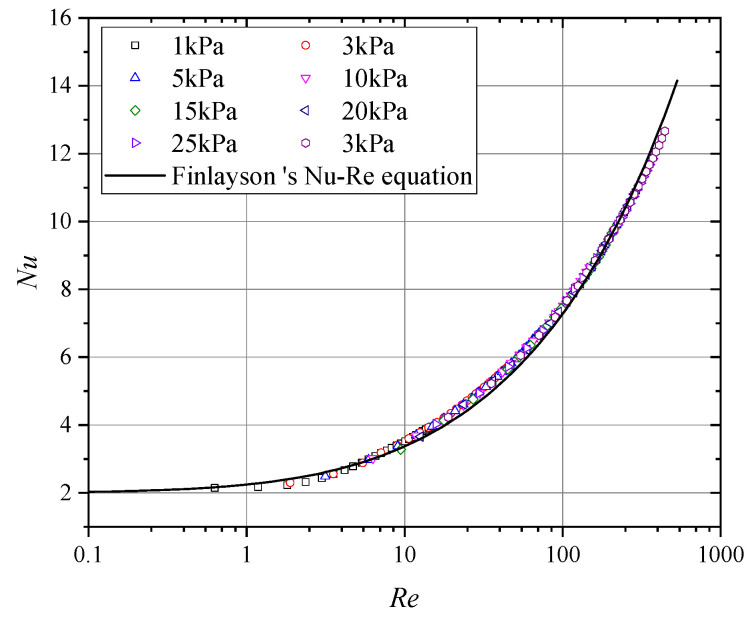
Nu-Re curves of the hot sphere.

In this equation, Pe represents the Peclet number, which is the product of the Reynolds number and the Prandtl number. The Prandtl number can be calculated based on temperature. As shown in Figure 13, the Nusselt numbers obtained from the experimental data are in close agreement with the theoretical Nusselt numbers, confirming the feasibility of using the constant heat flow hot sphere probe to characterize convective heat transfer under low-pressure conditions.

To analyze the probe’s signal response, the sensitivity of the probe to wind speed and pressure was calculated, as defined in Equation (12):

(12) $$s_{V}=\partial U/\partial V\;s_{P}=\partial U/\partial P$$

where $s_{V}$ and $s_{P}$ represent the probe’s sensitivity to wind speed and pressure, respectively; U is the probe’s output signal (mV), and V and P are the wind speed (m/s) and pressure (Pa), respectively.

**Figure 14:**
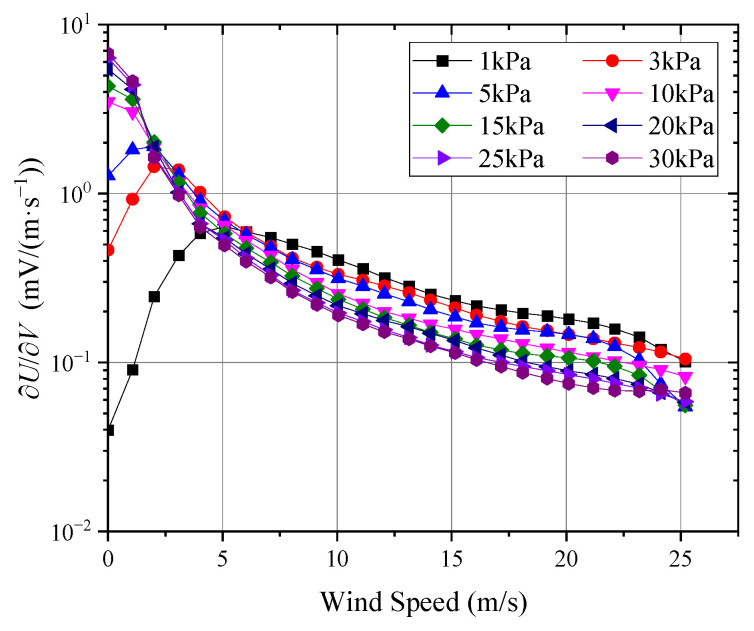
Probe sensitivity to wind speed.

**Figure 15:**
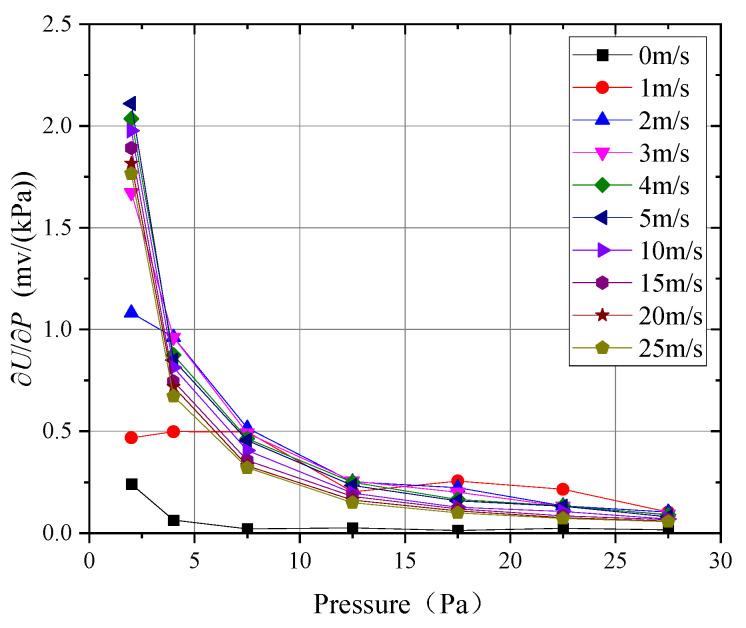
Probe sensitivity to pressure.

Figure 14 and Figure 15 show the probe’s sensitivity to wind speed $s_{V}$ and pressure $s_{P}$, as calculated using Equation (12).

As seen in Figure 14, when the ambient pressure was above 5 kPa, the probe’s sensitivity decreased monotonically with increasing wind speed. However, the effect of pressure on sensitivity showed opposite trends at wind speeds below and above 2 m/s. At wind speeds below 2 m/s, the probe exhibited greater sensitivity in higher-pressure environments, while, at wind speeds above 2 m/s, the probe’s sensitivity decreased with increasing pressure. This behavior was primarily related to the variation in the convective heat transfer coefficient; at higher pressures, the convective heat transfer coefficient increased rapidly with wind speed, resulting in higher sensitivity at lower wind speeds. However, the temperature of the thermal sphere quickly approached the flow field temperature, causing the signal to approach saturation more rapidly, thereby reducing sensitivity at higher wind speeds. When the ambient pressure was 5 kPa or lower, the probe’s sensitivity first increased and then decreased with wind speed, with the peak sensitivity occurring around 2 m/s, 3 m/s, and 5 m/s for pressures of 1 kPa, 3 kPa, and 5 kPa, respectively. At 1 kPa, the probe’s sensitivity was lowest near zero wind speed and slightly below its sensitivity at 25 m/s, but, above 5 m/s, its sensitivity exceeded that observed under the 3 kPa to 30 kPa conditions. As shown in Figure 15, at wind speeds above 2 m/s, the output signal varied with pressure in an almost exponential manner, whereas, at lower wind speeds, the output curve exhibited more nonlinear behavior.

**Figure 16:**
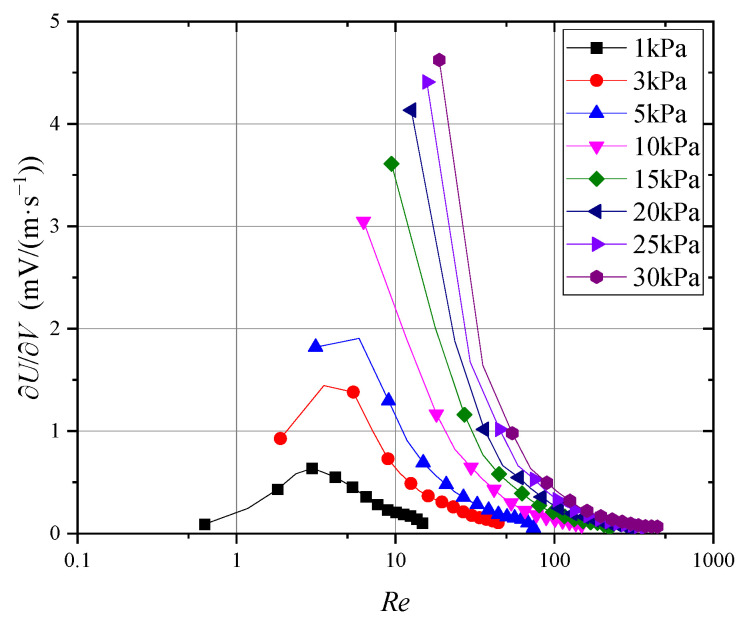
Wind speed sensitivity at different Reynolds numbers.

**Figure 17:**
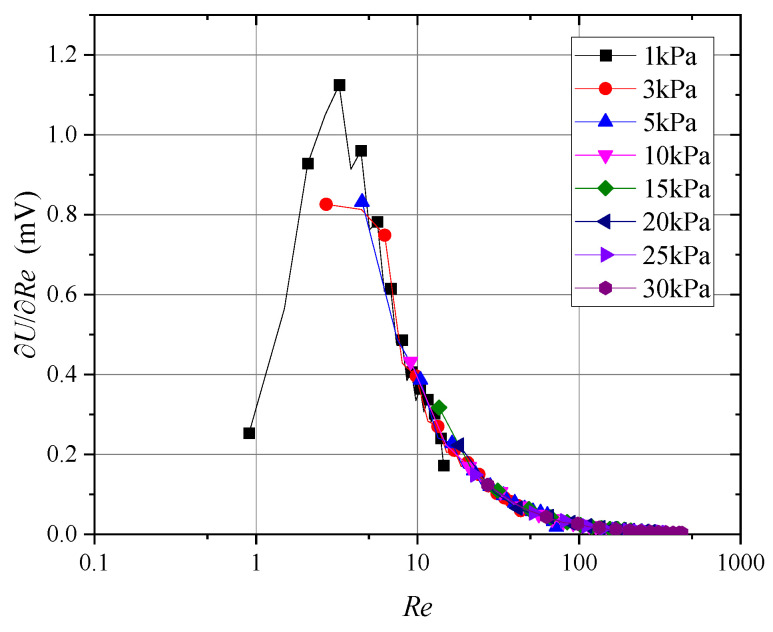
Probe sensitivity to Reynolds number.

Building on this, to assess the probe’s sensitivity across different Reynolds numbers, Figure 16 illustrates the wind speed sensitivity at varying Reynolds numbers. Furthermore, Figure 17 presents the derivative of the probe’s output signal with respect to the Reynolds number.

As shown in Figure 16, when the Reynolds number decreased to approximately 5, the probe’s wind speed sensitivity declined, which explains the nonlinear changes in sensitivity observed in Figure 14. Similarly, Figure 17 reveals substantial variability in the probe’s sensitivity at Reynolds numbers below 10, while the sensitivity became increasingly consistent as the Reynolds number exceeded 10. Therefore, for measurements at very low wind speeds or pressures, nonlinear variations in probe sensitivity must be carefully considered.

The wind speed measurement resolution could be calculated based on the voltage resolution of the acquisition equipment and the sensitivity of the probe in Figure 14:

(13) $$V_{NDC}=U_{NDC}/a$$

where $V_{NDC}$ is the wind speed resolution (m/s), $U_{NDC}$ is the voltage measurement resolution of the system (18 µV), and a is the sensitivity of the wind speed probe ($mV/(m\cdot s^{-1})$). It was determined that at 1 kPa, the wind speed measurement resolution at wind speeds between 5 m/s and 20 m/s could reach 0.1 m/s, indicating that measuring wind speed in a 1 kPa environment using a thermal anemometer probe is feasible for engineering applications.

**Figure 18:**
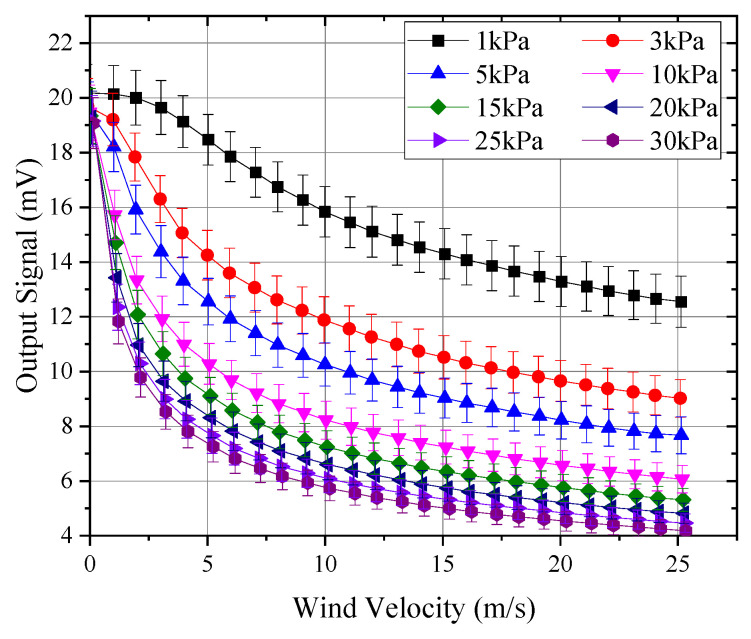
Standard error under different wind speeds.

**Figure 19:**
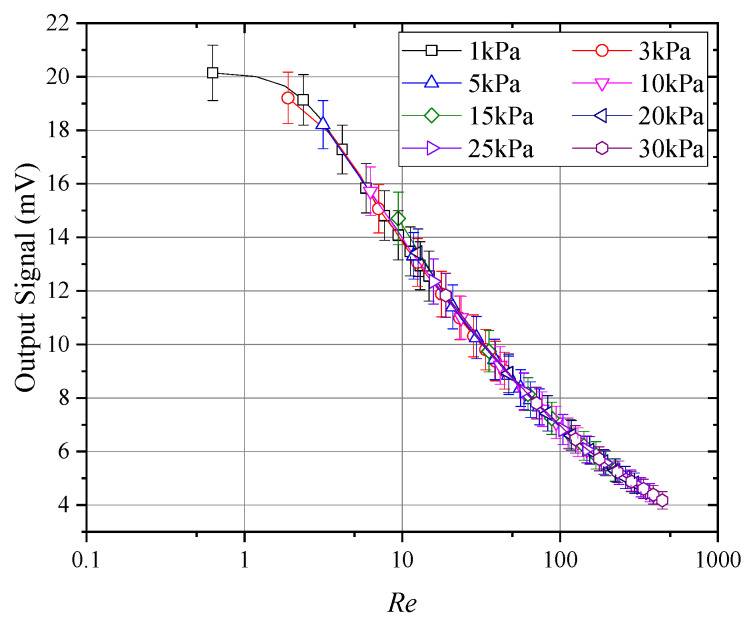
Standard error under different *Re*.

To statistically assess the individual variation characteristics of thermal sphere probes under different pressure conditions, Figure 18 shows the variation in the mean and standard deviation of probe signals as a function of wind speed across varying pressures. Similarly, Figure 19 depicts the changes in the mean and standard deviation of probe signals with Reynolds numbers (Re) under different pressure environments.

As seen in Figure 18 and Figure 19, the individual differences in probe output signals increased as the pressure decreased, with the standard deviation at 1 kPa being approximately three times that at 30 kPa. These variations primarily stemmed from the significant influence of viscous forces at low Reynolds numbers, where differences in the sensor’s microstructure and surface roughness notably affected the flow and heat transfer characteristics around the thermal sphere. As the Reynolds number increased, inertial forces emerged as the dominant factor, resulting in a gradual decrease in the impact of individual variations on the sensor’s surface. Additionally, at low pressures, the higher operating temperature of the thermal sphere exacerbated these signal differences. Therefore, for thermal anemometry at low pressures around 1 kPa, each probe must be individually calibrated; otherwise, individual differences between probes could result in significant wind speed measurement errors.

### 3.4. Wind Speed Calibration Method under Low Pressure

**Figure 20:**
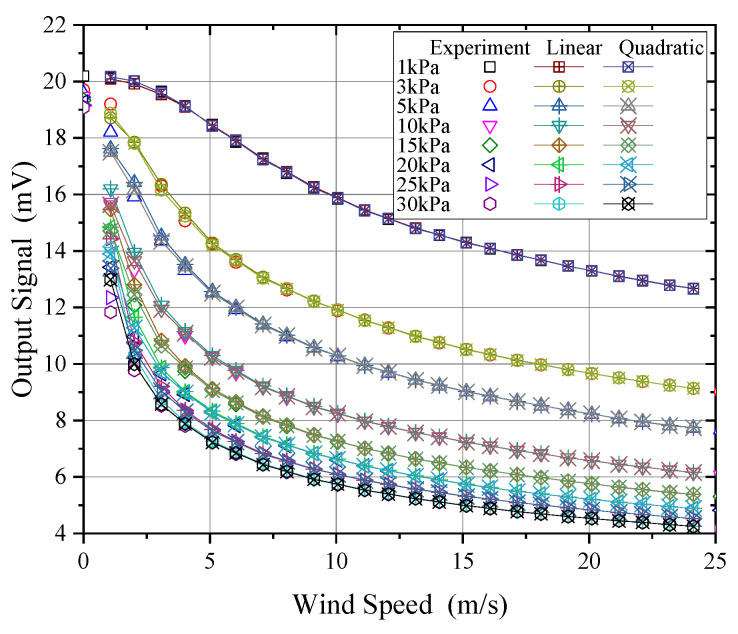
Wind speed–output signal interpolation.

**Figure 21:**
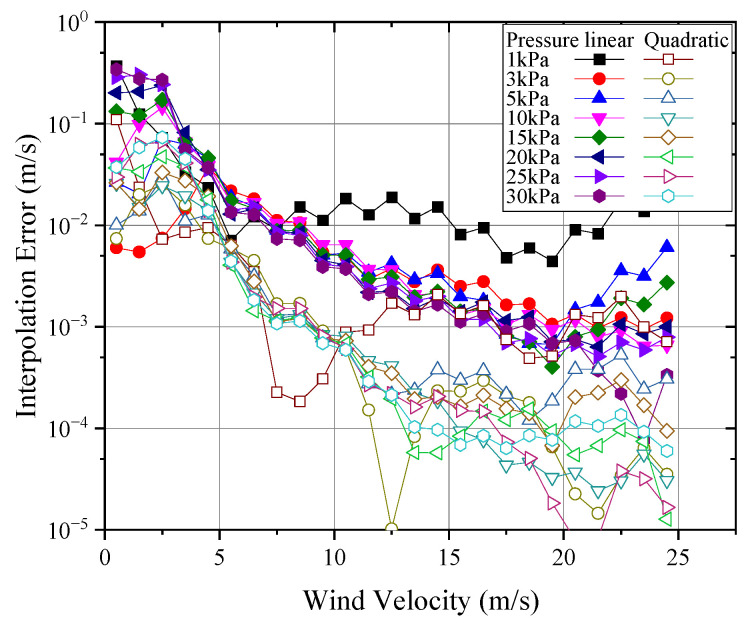
Maximum wind speed measurement error introduced by interpolation.

In the calibration of wind speed probes, it is only feasible to measure a limited number of data points, necessitating the use of piecewise interpolation for data application in real-world scenarios. Consequently, it is essential to evaluate the measurement error introduced by interpolation to select an appropriate calibration interval and interpolation method. Figure 20 presents the results of applying linear and quadratic interpolation methods to the wind speed–output signal functions at different pressures, with an interpolation interval of 1 m/s. As shown, both methods can capture the overall trend of the signal; however, at lower wind speeds, where the curve exhibits greater nonlinearity, linear interpolation may introduce significant errors. Based on the Lagrange interpolation remainder estimation Formula (10), the interpolation error can be estimated:

(14) $$\left|R_{n}(x)\right|\leq \frac{f_{max}^{\left(n+1\right)}(x)}{\left(n+1\right)!}\left|\omega_{n+1}(x)\right|$$

where $R_{n}(x)$ is the interpolation remainder; $f^{\left(n+1\right)}(x)$ is the maximum value of the n+1 derivative within the interpolation interval, which can be obtained by processing the original measured data using numerical differentiation; and $\omega_{n+1}(x)$ is a polynomial formed by the product of the differences between the interpolation point and the original data points, calculated based on the selected interpolation points. The maximum error introduced by piecewise interpolation is illustrated in Figure 21.

As shown in Figure 21, using quadratic interpolation for wind speed significantly reduced the maximum truncation error by an order of magnitude compared to linear interpolation. For wind speed measurements above 5 m/s at pressures between 1 kPa and 30 kPa, the fitting error could be kept below 0.05 m/s. However, for low wind speeds below 5 m/s, the fitting error was relatively large, approaching 0.5 m/s. In the 5 kPa to 30 kPa range, this was primarily due to the more nonlinear relationship between signal and wind speed within this range, leading to larger errors from fitting and interpolation. For the 1 kPa condition, the main reason was the lower sensitivity of the probe in this range, so even a small fitting error could result in significant wind speed measurement error. For the 3 kPa condition, the error mainly arose from the inflection point of the curve appearing around 2.5 m/s, making it difficult for quadratic fitting to accurately capture the trend of the curve below 5 m/s, resulting in instances where the local quadratic fitting error exceeded that of linear fitting.

Therefore, for wind speed calibration under low-pressure conditions, a calibration interval of 1 m/s can reduce interpolation error to within 0.05 m/s for wind speeds above 5 m/s. However, for conditions below 5 m/s, the calibration interval should be reduced to within 0.5 m/s. According to Equation (14), the maximum interpolation error can then be reduced to be within 0.05 m/s.

**Figure 22:**
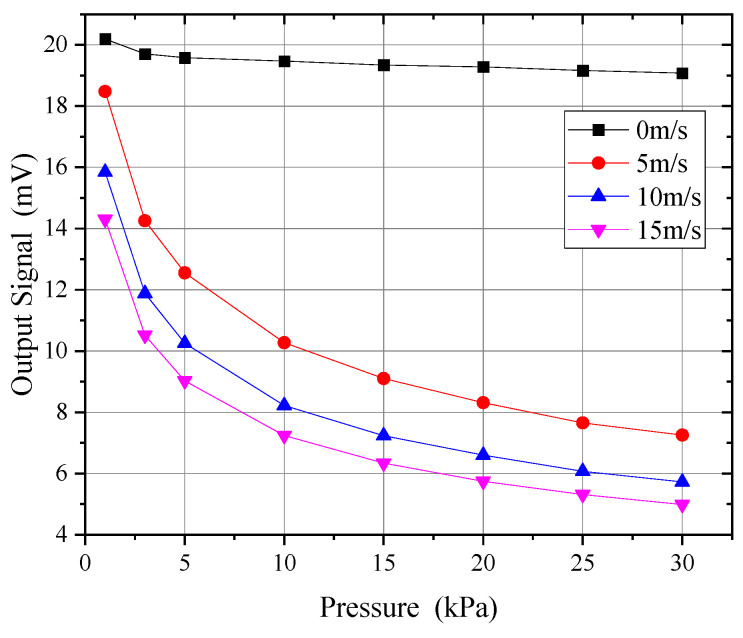
Effect of pressure on the measurement signal.

Figure 22 shows the effect of pressure on the measurement signal at wind speeds from 0 to 15 m/s. As can be seen, the effect of pressure on the wind speed signal was complex: at lower wind speeds, the impact of pressure was smaller and more linear, while at wind speeds above 5 m/s, the influence of pressure became more nonlinear.

**Figure 23:**
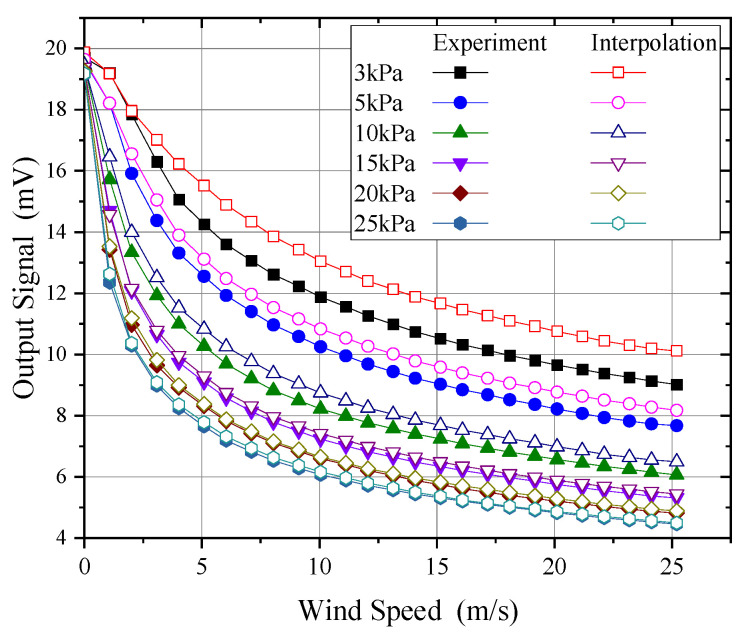
Result of linear interpolation of pressure.

**Figure 24:**
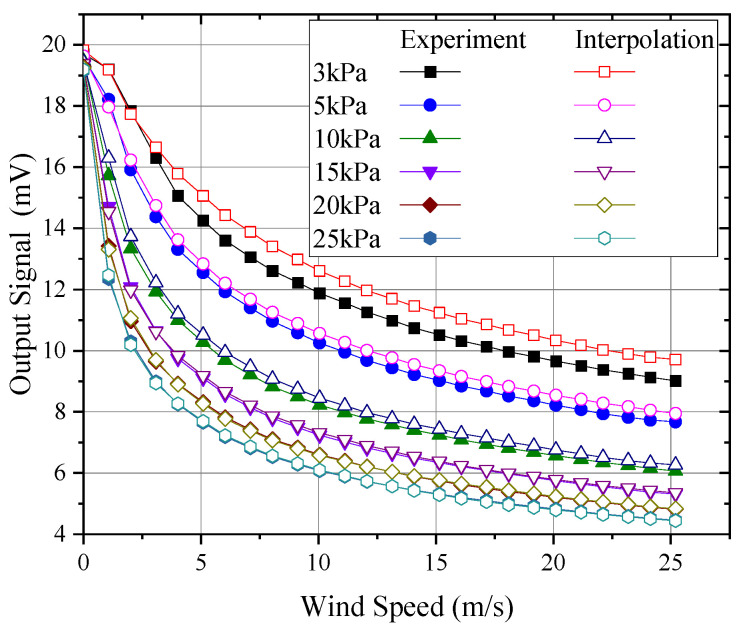
Result of quadratic interpolation of pressure.

Using experimental data, probe output under 3 kPa, 5 kPa, 10 kPa, 15 kPa, 20 kPa, and 25 kPa conditions was interpolated, with the results of linear and quadratic interpolation compared to measured data, as shown in Figure 23 and Figure 24, respectively.

**Figure 25:**
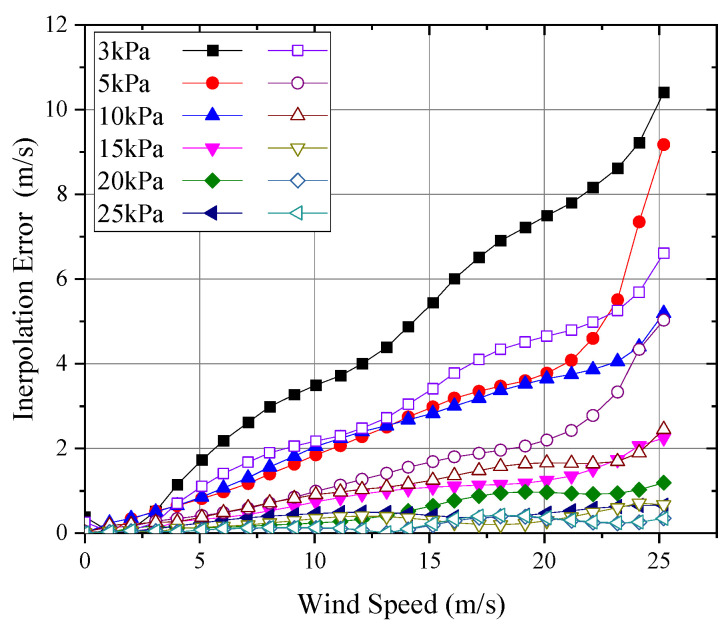
Comparison of linear and quadratic interpolation errors.

As shown in Figure 23 and Figure 24, both linear and quadratic interpolation struggled to accurately reflect the nonlinear effects of pressure, particularly under conditions below 3 kPa. Even with quadratic interpolation, significant discrepancies with the experimental data were observed. Figure 25 compares the wind speed measurement errors introduced by pressure interpolation. As shown, for pressures above 15 kPa, even with an interpolation interval of around 5 kPa, the wind speed measurement error due to pressure interpolation could be kept within 1 m/s. However, as the pressure decreased, the influence of pressure became increasingly significant. At 3 kPa, even using quadratic interpolation with data from 1 kPa, 5 kPa, and 10 kPa, errors exceeding 6 m/s could be introduced at higher wind speeds (>20 m/s). Therefore, for wind speed measurements below 10 kPa, it is essential to calibrate for each specific pressure to avoid significant measurement errors.

## 4. Discussion and Conclusions

To address the challenge of accurately characterizing wind speed under low-pressure conditions, where various wind speed probes struggle due to the thin air, this study focused on a typical constant-heat-flow hot-sphere anemometer. A wind speed simulation system based on the rotation method was constructed using a space environment simulation chamber and a high-precision turntable. Experimental research on the probe’s response characteristics in environments with pressures ranging from 1 kPa to 30 kPa was conducted. The Nu-Re analysis of the experimental data shows strong alignment with theoretical curves, affirming the validity of the experimental methodology employed in this study. Preliminary results indicate the feasibility of utilizing the hot-sphere anemometer for wind speed measurements in the stratosphere at altitudes up to 30 km. The conclusions are as follows:

1. Low pressure and low wind speed lead to a sharp decline in the Reynolds number of thermal anemometer probes, resulting in a gradual shift to nonlinearity in the output signal at these low Reynolds numbers. This nonlinearity significantly reduces wind speed measurement sensitivity in the range of 0 to 5 m/s, while sensitivity remains relatively stable for wind speeds exceeding 5 m/s. Thus, using a high-precision millivolt transmitter with a hot-sphere anemometer for measuring wind speeds at approximately 1 kPa pressure at 30 km altitude is feasible.
2. At extremely low Reynolds numbers, the influence of viscous forces in the fluid becomes more pronounced, leading to greater variations in flow and heat transfer characteristics due to differences in shape and surface roughness among probes from different batches. Consequently, at approximately 1 kPa, the individual discrepancies between thermal sphere probes can be up to three times greater than those observed at 30 kPa. Therefore, to meet the requirements for wind speed measurements under low-pressure conditions, it is essential to calibrate each probe individually, as extrapolation methods may introduce significant errors.
3. At around 1 kPa, the individual variations among hot-sphere anemometers can be up to three times greater compared to those at 30 kPa. Therefore, for wind speed measurement needs under low pressure, each probe should be individually calibrated, as extrapolation methods may introduce substantial errors.
4. Comparisons of linear and quadratic interpolation for the wind speed–output signal curves at different pressures indicate that the interpolation truncation error for wind speeds between 1 kPa and 30 kPa does not show significant differences. Quadratic interpolation reduces the wind speed fitting truncation error by an order of magnitude. To achieve a fitting error of 0.05 m/s, a calibration interval of 1 m/s is sufficient for wind speeds above 5 m/s, while, for speeds below 5 m/s, the calibration interval should be reduced to within 0.5 m/s.
5. Both linear and quadratic interpolation methods fail to adequately reflect the nonlinear impact of pressures below 10 kPa on the measurement signal. Therefore, for wind speed measurements in different low-pressure environments, it is advisable to calibrate each probe’s response individually at each wind speed to minimize the impact of pressure on measurement results.

## Figures and Tables

**Table 1:** Measurement equipment parameters.

	Parameter	Value
1	Range	0~75 mV
2	A/D resolution	12 bit
3	Power consumption	<1.5 W
4	Communication protocol	Modbus
5	Standard accuracy	±0.2% F.S
6	Temperature drift	±0.015%/℃
7	Response time	<0.03 s
8	Voltage influence	<0.1%

## Data Availability

The original contributions presented in this study are included in the article; further inquiries can be directed to the corresponding author.

## References

[1] d’Oliveira F.A., Melo F.C.L.D., Campos D.T. (2016). High-Altitude Platforms—Present Situation and Technology Trends. J. Aerosp. Technol. Manag..

[2] Gao W., Bi Y., Li X., Dong A., Wang J., Yang X. (2024). Variational Method-Based Trajectory Optimization for Hybrid Airships. Aerospace.

[3] Manikandan M., Pant R.S. (2021). Research and advancements in hybrid airships—A review. Prog. Aerosp. Sci..

[4] Weber P. Validation of a Thermal Management Concept for the Avionics Compartment of a Stratosphere HALE. Proceedings of the AIAA SCITECH 2024 Forum.

[5] Galvão J., Faria P., Mateus A., Pereira T., Fernandes S. (2021). Heatsinks to Cool Batteries for Unmanned Aerial Vehicles. Power Electron..

[6] Wang S., Wang H., Chang M., Xu J., Wang J., Yang X., Bai J. (2023). A novel battery thermal management system for an unmanned aerial vehicle using the graphene directional heat transfer structure. J. Power Sources.

[7] Oettershagen P. (2017). High-fidelity solar power income modeling for solar-electric uavs: Development and flight test based verification. arXiv.

[8] Li X., Lu F., Peng F., Fu W., Meng F., Man G. Thermal design and analysis of payload cabin of solar UAV in near space. Proceedings of the IOP Conference Series: Materials Science and Engineering.

[9] Zhu W., Li J., Xu Y. (2019). Optimum attitude planning of near-space solar powered airship. Aerosp. Sci. Technol..

[10] Zhang L., Li J., Wu Y., Lv M. (2019). Analysis of attitude planning and energy balance of stratospheric airship. Energy.

[11] Wilson C.F. (2003). Measurement of Wind on the Surface of Mars. Ph.D. Thesis.

[12] Spelay R.B., Adane K.F., Sanders R.S., Sumner R.J., Gillies R.G. (2015). The effect of low Reynolds number flows on pitot tube measurements. Flow Meas. Instrum..

[13] Alfonso-Corcuera D., Ogueta-Gutiérrez M., Fernández-Soler A., González-Bárcena D., Pindado S. (2022). Measuring relative wind speeds in stratospheric balloons with cup anemometers: The TASEC-lab mission. Sensors.

[14] Hanford A.D., Long L.N. (2006). The absorption of sound on Mars using the direct simulation Monte Carlo. J. Acoust. Soc. Am..

[15] Banfield D., Schindel D.W., Tarr S., Dissly R.W. (2016). A Martian acoustic anemometer. J. Acoust. Soc. Am..

[16] Bardera R., Sor S., Garcia-Magarino A., Gomez-Elvira J., Marin M., Navarro S., Torres J., Carretero S. (2018). Experimental and numerical characterization of the flow around the Mars 2020 rover. J. Spacecr. Rocket..

[17] Mallios S.A., Drakaki E., Amiridis V. (2020). Effects of dust particle sphericity and orientation on their gravitational settling in the earth’s atmosphere. J. Aerosol Sci..

[18] Merrison J.P., Gunnlaugsson H.P., Jensen J., Kinch K., Nørnberg P., Rasmussen K.R. (2004). A miniature laser anemometer for measurement of wind speed and dust suspension on Mars. Planet. Space Sci..

[19] Rodriguez-Manfredi J.A., De la Torre Juárez M., Alonso A., Apéstigue V., Arruego I., Atienza T., Banfield D., Boland J., Carrera M., Castañer L. (2021). The Mars Environmental Dynamics Analyzer, MEDA: A Suite of Environmental Sensors for the Mars 2020 Mission. Space Sci. Rev..

[20] Crisp D., LaBaw C., Mahoney C., Serviss O., Harri A.-M., Polkko J., Calcutt S., Tillman J.E., Larsen S., Haberle R. Netlander Atmis wind and temperature instruments. Proceedings of the International Workshop on Mars Atmosphere Modelling and Observations.

[21] Numata D., Anyoji M., Sugino Y., Nagai H., Asai K. Characteristics of thermal anemometers at low-pressure condition in a mars wind tunnel. Proceedings of the 49th AIAA Aerospace Sciences Meeting Including the New Horizons Forum and Aerospace Exposition.

[22] Li X., Yin X., Gao Q., Li Q., Wang J. Simulation Research on Hot Bulb Anemometer Under Low Pressure. Proceedings of the 3rd International Conference on Electromechanical Control Technology and Transportation (ICECTT 2018).

[23] Quix H., Quest J., Brzek C. Hot-wire measurements in cryogenic environment. Proceedings of the 49th AIAA Aerospace Sciences Meeting Including the New Horizons Forum and Aerospace Exposition.

[24] Bardera-Mora R., Garcia-Magariño A., Sor S., Urdiales M. LDA characterization of the Mars 2020 rover influence on the wind measurements at low Reynolds. Proceedings of the 2018 Applied Aerodynamics Conference.

[25] Pezzella G., Viviani A. (2020). Mars Exploration, a Step Forward.

[26] Domínguez-Pumar M., de la Torre Juárez M., Navarro S., Marin M., Gomez-Elvira J., Rosero-Pozo C., Manyosa X., Bermejo S., Rodríguez-Manfredi J.A. (2024). Dynamics of constant temperature anemometers for the Martian Atmosphere. Measurement.

[27] Kowalski L., Atienza M.T., Gorreta S., Jimenez V., Dominguez-Pumar M., Silvestre S., Castañer L.M. (2015). Spherical wind sensor for the atmosphere of mars. IEEE Sens. J..

[28] Nauk A. (2007). Implementation of velocity slip and temperature jump boundary conditions for microfluidic devices. Pr. Inst. Podstawowych Probl. Tech. Pan.

[29] Wen G., Boying L., Dongliang W., Xiyuan L., Dongxing T., Xiaoning L., Xiaoning Y. (2024). Numerical simulation of plume flow field in vacuum chamber based on slip flow model. Spacecr. Environ. Eng..

[30] Xiyuan L.I., Yaqin H., Qinghua G., Lina Z., Jing W. (2018). The heat transfer model of hot-sphere anemometer in Martian atmospheric environment and corresponding test validation. Spacecr. Environ. Eng..

[31] Xiyuan L., Xiaofang Y., Yaqin H., Qinghua G. Research on applicability of hot-bulb anemometer under low pressure. Proceedings of the MATEC Web of Conferences.

[32] Merrison J., Gunnlaugsson H.P., Kinch K., Jacobsen T., Jensen A., Nørnberg P., Wahlgreen H. (2006). An integrated laser anemometer and dust accumulator for studying wind-induced dust transport on Mars. Planet. Space Sci..

[33] Anyoji M., Nagai H., Asai K. Development of low density wind tunnel to simulate atmospheric flight on Mars. Proceedings of the 47th AIAA Aerospace Sciences Meeting Including the New Horizons Forum and Aerospace Exposition.

[34] Wilson C., Camilletti A., Calcutt S., Ligrani P. (2008). A wind tunnel for the calibration of Mars wind sensors. Planet. Space Sci..

[35] Haihua Y., Zejing C., Xingang L., Jianxun R. (2003). Measurement of Low Wind Velocity under Low Pressure by an Improved Hot-bulb Probe. Space Med. Med. Eng..

[36] Djanali V., Syah A.N., Rizal S. (2016). Numerical study of mixed convection around a heated circular cylinder. Appl. Mech. Mater..

[37] Finlayson B.A., Olson J.W. (1987). Heat transfer to spheres at low to intermediate Reynolds numbers. Chem. Eng. Commun..

